# Thromboembolic Events in Patients with Inflammatory Bowel Disease: A Comprehensive Overview

**DOI:** 10.3390/diseases10040073

**Published:** 2022-09-30

**Authors:** Dhir Gala, Taylor Newsome, Nicole Roberson, Soo Min Lee, Marvel Thekkanal, Mili Shah, Vikash Kumar, Praneeth Bandaru, Vijay Gayam

**Affiliations:** 1 American University of the Caribbean School of Medicine, 1 University Drive at Jordan Dr, Cupecoy, Sint Maarten, The Netherlands; 2 Department of Internal Medicine, The Brooklyn Hospital Center, 121 DeKalb Ave, Brooklyn, NY 11201, USA; 3 Department of Gastroenterology, The Brooklyn Hospital Center, 121 DeKalb Ave, Brooklyn, NY 11201, USA

**Keywords:** inflammatory bowel disease, thromboembolism, Crohn’s disease, ulcerative colitis, deep vein thrombosis, pulmonary embolism

## Abstract

Inflammatory bowel disease (IBD), Crohn’s disease and ulcerative colitis are chronic inflammatory disorders of the intestines. The underlying inflammation activates the coagulation cascade leading to an increased risk of developing arterial and venous thromboembolic events such as deep vein thrombosis and pulmonary embolism. Patients with IBD are at a 2–3-fold increased risk of developing thromboembolism. This risk increases in patients with active IBD disease, flare-ups, surgery, steroid treatment, and hospitalization. These complications are associated with significant morbidity and mortality making them important in clinical practice. Clinicians should consider the increased risk of thromboembolic events in patients with IBD and manage them with appropriate prophylaxis based on the risk. In this review, we discuss the literature associated with the pathophysiology of thromboembolism in patients with IBD, summarize the studies describing the various thromboembolic events, and the management of thromboembolism in patients with IBD.

## 1. Introduction

Inflammatory Bowel Disease (IBD) is characterized by chronic inflammation of the intestines resulting from an interplay between environmental and genetic factors. The two main types of IBD are Crohn’s disease and ulcerative colitis. While Crohn’s disease and ulcerative colitis both present with many of the same symptoms, including persistent diarrhea, abdominal pain, weight loss, and fatigue, they differ in that Crohn’s disease may affect any part of the digestive tract, whereas ulcerative colitis specifically affects the large intestine [1]. The prevalence of IBD is highest in Europe and North America with the prevalence of IBD in North America exceeding 0.3%. However, the incidence of IBD is beginning to stabilize in these regions and increase in other newly industrialized areas, such as Brazil and Taiwan, as they are becoming more Westernized [2]. In the USA, the incidence of Crohn’s disease is higher in African Americans and whites, while ulcerative colitis is more common in Mexican Americans. Additionally, African Americans have a higher incidence of sequelae, such as IBD-associated arthritis (*p* = 0.004), and ophthalmological manifestations, such as uveitis (*p* = 0.028) from Crohn’s disease than whites [3]. The most common age for onset of IBD is 20–30 years old, but those with Crohn’s disease have a mean age of diagnosis that is 5–10 years earlier than those with ulcerative colitis [4].

IBD presentation is very general and may overlap with other syndromes or diseases, such as irritable bowel syndrome. Common symptoms include diarrhea (with or without blood), constipation (especially with ulcerative colitis), painful bowel movements, abdominal pain (right lower quadrant for Crohn’s and periumbilical for ulcerative colitis), as well as nausea and vomiting [5]. Several environmental factors play a role in IBD, including smoking [6] and diet, with a Western diet, associated with low fiber, high sugar, and fatty foods, contributing to an increase in IBD incidence [7]. Additionally, the hygiene hypothesis posits that when children are raised in a highly hygienic environment, they are not exposed to organisms that help build the immune system. Therefore, this environment leads to an underdeveloped immune system which may result in IBD later in life, as this may be caused by an inappropriately large immune response to the contents of the intestines. Indeed, in many underdeveloped nations where children are exposed to intestinal helminths, the incidence of IBD is much lower than in countries where children are exposed to very hygienic environments that lack exposure to intestinal helminths [8].

Many complications are associated with IBD including many extraintestinal manifestations [9]. These complications include musculoskeletal system, dermatologic and oral systems, hepatopancreatobiliary system, ocular system, metabolic system, and renal system complications [9]. Joints, skin, and eyes are most commonly affected with manifestations such as peripheral arthritis, episcleritis, or erythema nodosum [10]. While it is important to keep in mind common complications of IBD, rare complications are also important to note. One rare complication associated with IBD is thrombosis.

## 2. Thrombosis

Thrombosis is the result of the coagulation cascade in which a fibrin clot forms in a vein or artery. Thrombosis is a normal response to endothelial injury and contributes to the healing response; however, thrombosis can also lead to myocardial infarction, pulmonary embolism (PE), and deep vein thrombosis (DVT), among other conditions [11]. The coagulation pathway has four main events: vessel constriction to limit blood flow to the site of injury, platelet activation to form the initial platelet plug, formation of the fibrin clot, and fibrinolysis to remove the clot as wound healing is completed. Any defects in the clotting cascade can lead to abnormal blood clots in the body leading to various pathologies [12]. The cascade is initiated by both the intrinsic (internal stimuli) and extrinsic (external stimuli/trauma) pathways. Both pathways converge on Factor X and continue through the cascade to ultimately form thrombin which provides positive feedback to continue clot formation [13]. Thrombin also converts fibrinogen to fibrin which forms the cross-linked fibrin clot, the end product of the cascade [12]. Many of the factors in the cascade are serine proteases, which can be inactivated by anticoagulants, such as antithrombin, a serpin that irreversibly inactivates serine proteases, which targets Factor Xa and thrombin. Heparin, an anticoagulant used to treat venous thrombosis stimulates antithrombin activity and thus accelerates the inactivation of various clotting factors [12].

While coagulation is part of a normal response to endothelial injury, various factors can predispose someone to venous thrombosis, which are described in Virchow’s Triad: endothelial injury, stasis, and a hypercoagulable state [14]. Endothelial injury, resulting from smoking, chronic hypertension, or atherosclerosis, creates turbulent blood flow leading to thrombosis in the area of damage. Stasis of blood, common in bedridden patients, can interfere with the interaction between natural anticoagulant molecules and surface proteins leading to a thrombus. Lastly, a hypercoagulable state, seen in pregnancy, oral contraceptive use, and cancer, can also lead to resistance to natural anticoagulant molecules causing thrombus formation. Thus, these three factors can lead to abnormal thrombosis caused by various disease processes which also puts these patients at risk for thromboembolism and other complications [14,15].

## 3. Pathophysiology and Risk Factors for Thrombosis

### 3.1. Introduction

**Figure 1:**
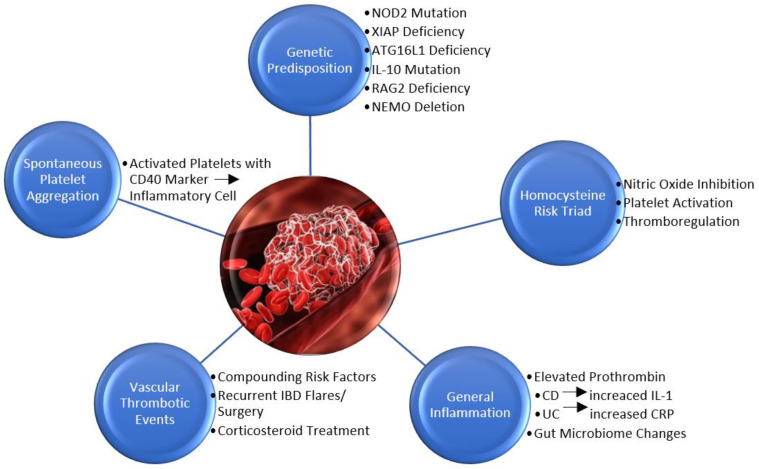
Summary of risk factors contributing to thrombosis in IBD.

The underlying mechanism for disease in IBD is chronic inflammation. Research is not yet conclusive of whether the altered gut microbiota is a cause or effect of the inflammation leading to IBD; however, it is known to play a role in the pathophysiology of disease. The decreased diversity of intestinal bacteria species and decreased quantity of anti-inflammatory bacteria *Faecalibacterium prausnitzii* are known predictive factors [16]. The intestinal microbiota is located within the endothelial cells and normally enhances mucus secretion for digestion and promote fiber fermentation. Alteration of the microbiota, therefore, disrupts natural digestion processes and promotes inflammation via endothelial damage [17]. The pathophysiology of inflammation leading to IBD can be caused by genetic or biological factors combined with a self-immune response (Figure 1). Increased inflammation furthermore initiates the coagulation cascade leading to a higher risk of thrombosis.

### 3.2. Genetic Predisposition

A recent study used mouse models to test genetic mutations associated with IBD to include nucleotide-binding oligomerization domain-containing protein 2 (NOD2), ATG16L1, recombination activating gene 2 (RAG2), interleukin 10 (IL-10) receptor deficiency, and nuclear factor kappa beta (NF-κB) essential modulator (NEMO) [18]. NOD2 negatively regulates toll-like receptors to inhibit the NF-κB signaling for immune response and anti-inflammatory release of IL-10 [19,20]. Mutations in NOD2 thus lead to decreased immune regulation and increased intestinal inflammation. NOD2 mutations are also associated with decreased anti-inflammatory *Faecalibacterium* and increased infectious *Escherichia* species in the microbiota further leading to inflammation [21,22,23]. Studies suggest that NOD2 mutations lead to defective bacterial phagocytosis resulting in a heightened immune response necessary to compensate [24]. It is further hypothesized that NOD2 is involved in antimicrobial peptide (AMP) expression and production in secretory Paneth cells of the small intestines. Defects in Paneth cells eliminate one of the major immunomodulating elements in the small intestines and lead to increased intestinal inflammation [25]. Homozygous mutations of NOD2 are, therefore, associated with a 20-fold increased risk for Crohn’s Disease [26]. Later studies have identified X-linked inhibitor of apoptosis (XIAP) deficiencies to be early indicators of IBD due to NOD2’s dependency on XIAP to complete an immune response [27,28]. Although most research focuses on IBD in adults, genome wide association studies (GWAS) support IL-10 receptor mutations are correlated with pediatric IBD and neonatal onset IBD. The mutation inhibits IL-10 from regulating tumor necrosis factor (TNF-α), thus promoting a pro-inflammatory state in infants and children [29].

ATG16L1 deficiencies lead to inflammation through a similar mechanism. Defective ATG16L1 genes decrease microbial autophagy and require the immune response to heighten in compensation [30]. Gene deficiency is also associated with decreased antimicrobial activity and expression of cell defensin proteins HD5 and HD6 of Paneth cells [31]. The mutation furthermore is associated with an increase in *Bacteriodes fragilis* in the microbiota. *B. fragilisis* is naturally a commensal bacteria that are commonly decreased in the microbiota of patients with IBD. In the T300A variant of ATG16L1 mutations, however, *B. fragilis* is increased in the microbiota but is pro-inflammatory by inhibiting T-lymphocyte development [32]. ATG16L1 deficiencies thus result in intestinal inflammation associated with IBD. Khan et al. concluded that a RAG2 deficiency in mice also leads to chronic colitis indicative of IBD [33]. RAG2 deficiencies prevent correct VDJ recombination of lymphocytes. The deficiency thus inhibits the maturation of B and T cells and results in the overactivation of cytokine and chemokine response. The induced cytokine storm causes a domino effect of immune cell activation which triggers inflammatory factors. Some of the inflammatory induced factors include interferon gamma (INF-γ), TNF, IL-1, IL-6, IL-17, IL-18, and Janus kinase signal transduction and activator of transcription (JAK-STAT3) [34]. Cytokine storm activation, therefore, causes systemic inflammation correlated with IBD. NF-κB, although normally part of the pro-inflammatory pathway, may have protective factors for intestinal epithelial cells. Indeed, a study on mice with conditionally ablated NEMO, essential for NF-κB activation, developed intestinal inflammation leading to epithelial cell apoptosis and translocation of microbes into the mucosa [35].

### 3.3. Inflammation’s Role in Thrombosis

Biological factors leading to IBD include a variety of elements causing increased inflammation which is a known initiator of the coagulation cascade. A study in 1995 concluded patients with Crohn’s Disease and Ulcerative Colitis often have increased thrombin levels leading to thrombosis. The study used prothrombin fragments 1 and 2 (F1 + 2) and thrombin–antithrombin III complex (TAT) as markers to identify thrombin. The increased thrombin is due to the release of TNF and IL-1 inflammatory response initiating tissue factor (TF) to begin the coagulation cascade. The anticoagulation activity of endothelial thrombin and protein C is suppressed simultaneously. The study revealed Crohn’s Disease was correlated with coagulation initiated by increased IL-1 levels, whereas Ulcerative Colitis was correlated with increased levels of C-reactive protein (CRP) [36]. CRP initiates the inflammatory response through IL-6 and IL-8 activation [37]. Thompson et al. validated the correlation between inflammatory-induced coagulation and IBD by reporting a decreased frequency of IBD in patients with Hemophilia or Von Willebrand Disease. Such patients have a deficiency of von Willebrand factor and factors VIII and XI of the coagulation cascade resulting in decreased thrombosis [38].

Another factor that leads to an increased risk of thrombosis in IBD includes changes in the gut microbiome which activates an inflammatory response and initiates coagulation. The use of antibiotics, specifically metronidazole, fluoroquinolones, and quinolones, is reported to have a strong association with the development of Crohn’s Disease. A study reported, furthermore, that antibiotic use in the first year of life leads to an increased risk of developing IBD as a child [39].

### 3.4. Homocysteine Risk Triad

Further reports indicate an increase in the amino acid homocysteine also induces an inflammatory response in patients with disorders such as IBD, systemic lupus, rheumatoid arthritis, and multiple sclerosis. A leading cause of hyperhomocysteinemia is folate and vitamin B deficiency. Folate and vitamin B are cofactors required for the catabolism of homocysteine and vitamin deficiencies thus result in increased serum and mucosal homocysteine levels [40]. Inflammatory-induced malabsorption in the intestines and dietary restrictions used to treat IBD lead to a strong correlation with hyperhomocysteinemia. The mechanism of homocysteine-induced intestinal endothelial cell inflammation is due to vascular cell adhesion protein-1 (VCAM-1) upregulation, monocyte chemoattractant protein-1 (MCP-1) production, and p38 phosphorylation [41,42].

Homocysteine is known to cause inflammation through alternative mechanisms as well. Increased homocysteine inhibits nitric oxide (NO), thus inhibiting vasodilation. NO production is inhibited through increased production of reactive nitrogen and oxygen species [43,44] and a deficiency of common methyl donor s-adenosyl-methionine (SAM) preventing DNA methylation [45]. Increased homocysteine also inhibits thromboregulation factors by inhibiting protein C and thrombomodulin through the reduction of disulfide bonds on an epidermal growth factor domain [46]. A reduction of antithrombin activity is another mechanism of thromboregulation inhibition [47]. Increased homocysteine further leads to thrombosis through platelet activation due to an increase in factor V [48], thromboxane A2 [49,50], a three-fold increase in arachidonic acid peroxidation product 8-iso-prostaglandin F2α, [51] increasing TF, a cofactor for coagulation factor VII, and mRNA synthesis [52].

### 3.5. Venous Thrombotic Events

Studies of East Asian and Mediterranean populations concluded that women with IBD have an increased risk of venous thromboembolism (VTE) due to compounding risk factors such as hormone replacement therapy, oral contraceptives, and pregnancy. Oral contraceptives containing estrogen lead to increased production of coagulation factors, increasing VTE three to six-fold [53]. Pregnancy increased the risk of VTE in women five- to six-fold by simultaneously increasing fibrinogen production and decreasing the anticoagulant protein S [54].

Two studies of Asian populations, East Asia and Korea, concluded patients with IBD have a two-fold risk of VTE [55,56]. Asian populations have a significantly lower incidence of VTE in comparison to Western nations; the increased prevalence in patients with IBD, therefore, shows a strong correlation. The Korean study indicates a 27-fold risk of VTE during hospitalizations associated with IBD flares. Additionally, they noted a 40-fold risk of VTE during the postoperative stages of IBD-related bowel resection [56]. The study in East Asia indicated that 54% of patients with IBD and VTE had a surgical history [55]. The intestinal area attacked by IBD is more sensitive to inflammatory response due to the immense commensal bacterial population. Alteration of the gut microbiome due to chronic stress, surgery, or IBD attacks leads to inflammation and activation of the coagulation cascade leading to thrombosis. Furthermore, one of the primary treatments of IBD is corticosteroids which induce coagulation through an increase in factors VII, VIII, and IX [57]. Indeed, a study reported an approximately five-fold increase in the risk of VTE in patients receiving corticosteroid therapy for IBD [58].

### 3.6. Spontaneous Platelet Aggregation

Uniquely, studies indicate platelets are 30% more likely to spontaneously aggregate in patients with IBD regardless of disease severity and clinical activity [59]. In patients with IBD, platelets circulate in an activated state identified by P-selectin, GP53, β-thromboglobulin, and CD40 ligand (CD40L) markers [60,61]. The activated platelet markers in Crohn’s Disease are more prevalent in capillaries indicative of platelet concentration and eventual thrombosis in the intestinal microcirculation [62].

Due to the presence of CD40L on activated platelets in patients with IBD, their platelets themselves are considered inflammatory cells [63]. The CD40L positive platelets can adhere to mucosal microvascular endothelium in the intestines to initiate an inflammatory response [64]. The mechanism of platelet inflammatory response is through upregulation of VCAM-1 and intercellular adhesion molecule-1 (ICAM-1) to secrete IL-8 and attract neutrophils. The platelets also attract monocytes and memory T-cells via chemokine RANTES [65]. In addition to containing CD40L surface markers, the activated platelets also express CD40 surface markers to activate platelets and recruit T-cells during intestinal inflammatory responses [64].

## 4. Thromboembolic Events in Patients with IBD

### 4.1. Introduction

**Figure 2:**
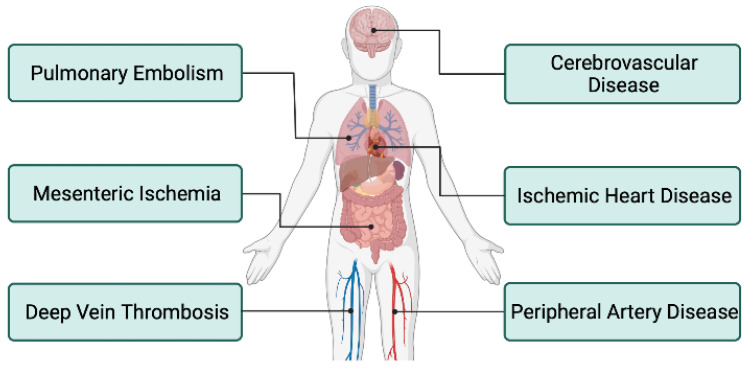
Arterial and venous thrombosis associated with IBD.

Patients with IBD are at an increased risk for arterial and venous thromboembolic events (Figure 2). The most common ones are VTE and PE. Arterial thromboembolism (ATE) is less common than VTE in patients with IBD. However, numerous case reports and case series have reported ATE in patients with IBD. ATE may involve thrombosis and/or occlusion of the cerebral [66,67], splanchnic [68], carotid [69], coronary [70], aorta [71], renal, and upper and lower extremity [72] arteries. Incidence is more common after interventional procedures, however, can occur spontaneously.

### 4.2. Deep Vein Thrombosis and Pulmonary Embolism

Over the past decade, multiple studies have focused on defining the association of IBD with the risk of VTE and have discussed the epidemiological and clinical features of VTEs in patients with IBD [73,74].

Various studies have looked at the incidence and risk of VTE in patients with IBD compared to the general population [75,76,77,78]. A few studies have compared the risk of VTE between hospitalized patients with IBD to patients without IBD [79,80,81]. Additionally, studies were done on more selective populations such as the risk of VTE in pregnant females with IBD [82] and the risk of VTE in postoperative patients with IBD [83]. Lastly, one study evaluated the risk of recurrent DVTs in adult patients with IBD [84] (Table 1).

In two meta-analyses that analyzed the overall risk of VTEs, DVT, and PE, it was found that there is an approximately 2-fold increased risk for VTEs in patients with IBD. [73,85]. The first study reported an approximately two-fold significantly increased risk of VTE in patients with IBC compared to the general population (RR = 2.20; 95% CI 1.83–2.65) [85]. Similarly, another meta-analysis study reported that patients with IBD were at a significantly increased risk for developing VTE compared to the general population (RR = 1.96; 95% CI: 1.67–2.30) with no difference between UC and CD [73].

A study by Papay et al. evaluated a total of 2811 patients with IBD reporting the incidence and prevalence of VTE and other related clinical features in this cohort [86]. This study reported an incidence and prevalence of all VTEs to be 6.3/1000 person-years and 5.6% (157/2811), respectively. The most commonly reported VTE included DVT and/or PE (about 90%). The other rare locations of VTE reported were the portal, the superior mesenteric, the splenic, the internal jugular, and the cerebral veins. Additionally, there was no significant difference in the frequency of all VTEs when comparing patients with CD to patients with UC. However, patients with CD had a higher prevalence and incidence of DVT and/or PE [86].

VTEs occur at a younger age in patients with IBD compared to the general population. Bernstein et al. analyzed data from hospitalized patients with and without IBD and found that hospitalized patients with IBD had a higher risk of VTE, DVT, and/or PE than those in the general population [79]. In patients younger than 40 years old, the most noticeable difference was observed. The incidence ratio rate for VTE was 4.5 for patients with UC and 9/6 for patients with CD compared to those in the general population. [79].

Patients with IBD are at a greater risk of morbidity and mortality from thromboembolic complications compared to the general population [87]. There is an increased risk of developing postoperative VTE in patients with IBD [88]. Indeed, a national study reported an increased risk of postoperative DVT and PE in patients with IBD undergoing intestinal (OR = 2.03; 95% CI, 1.52–2.70) or non-intestinal surgery (OR = 4.45; 95% CI, 1.72–11.49) [89].

Patients with IBD have a significantly higher risk for recurrent VTE compared to the general population. Indeed, a study reported the recurrence of thromboembolic events to be about 10–13% in patients with IBD [90]. Novacek et al. compared the risk of recurrent VTE in patients with and without IBD 5 years post discontinuation of anticoagulants reporting a statistically significant higher risk in patients with IBD (hazard ratio = 2.5; 95% CI: 1.4–4.2; *p* = 0.001) [84]. Additionally, a study found IBD to be an independent risk factor for recurrent VTE with a relative risk of 2.5 after controlling for activity status [84]. Similarly, another study reported an incidence of recurrent VTE to be 25% in patients with IBD. Additionally, the majority of the VTE was found to be the same type as DVT or PE and in the same location as the first episode [86].

The frequency of thromboembolic events is higher in patients with active IBD and correlates directly with the extent and severity of the diseases. Additionally, most thromboembolic events occur in the absence of provoking factors [42,90,91]. A study found that 74% of first-time VTE was unproved in patients with IBD [84]. Additionally, IBD complicated by stenosis, abscess or fistulas, use of corticosteroids, and recent hospitalizations for IBD flares were all associated with increased risk for VTEs [80].

Solem et al. reported that 80% of patients with IBD (both CD and UC) had active disease at the time of VTE. Additionally, 76% of the UC patients had concurrent pancolitis and 79% of CD patients had concurrent colonic involvement [91]. In contrast to the above, Talbot et al. found that VTEs occurred when the disease was in remission in almost 30% of patients and that peripheral VTEs occurred spontaneously in 77% of patients [90].

Studies have found hospitalizations associated with IBD flares to have significantly increased the risk of VTE. Grainge et al. reported a threefold increased relative risk of VTE in hospitalized patients with IBD compared to controls [78]. Similarly, Nguyen and Sam reported a significantly higher risk of VTE in patients with IBD (OR 1.85 for UC and OR 1.48 for CD). Additionally, they found VTE was associated with increased mortality, longer hospital stays a higher healthcare cost (OR 2.5) [80]. Indeed, other studies have confirmed the higher risk of VTE in all hospitalized patients with IBD secondary to non-flare-up, flare-up, or surgery [92].

### 4.3. Peripheral Artery Disease

The literature has mixed evidence for the risk of peripheral artery disease (PAD) associated with IBD. Two meta-analyses with two studies each reported no significant increase in the risk of PAD in patients with IBD [73,93]. On the contrary, Lin et al. reported a significantly increased risk of PAD in patients with IBD after adjusting for age, sex, and comorbidities (HR 1.24). Additionally, the risk for PAD was highest in patients with more than two annual IBD-related medical hospitalizations (HR 27.5) [94]. These findings suggest an association of disease severity with the risk of PAD.

Kirchgesner et al. reported a significantly higher risk of PAD in patients with IBD compared with the general population (SIR 1.27). Interestingly, they found a significant increase in the risk in patients with CD (SIR 1.65) but not with UC (SIR 1.07) [95]. Additionally, they noted that the risk was highest in patients with CD who were younger than 35 (SIR 3.04). Indeed, this risk decreased with increasing age, eventually, becoming non-significant in patients older than 75 years [95], suggesting an association between age and risk of PAD.

### 4.4. Cerebrovascular

While IBD manifests as an inflammatory disease in the colon, it can also be accompanied by disorders outside of the colon, including nervous system manifestations, such as cerebrovascular disease [96]. Many studies have investigated the link between IBD and cerebrovascular disease, with the majority detailing that those with IBD have an increased risk of cerebrovascular disease; however, there are some differences between those with ulcerative colitis and those with Crohn’s disease [97,98]. While 33% of patients with IBD have extraintestinal manifestations, cerebrovascular disease is reported in only 3% of patients with IBD; however, it is still an important consideration, as some therapies may increase the risk of neurologic manifestations [99].

In a population-based cohort study analyzing 20,795 patients with IBD matched to 199,978 controls, Kristensen et al. aimed to identify differences between disease activity (IBD overall, persistent IBD, and IBD in remission) and risk of MI, stroke, and cardiovascular death. The study found that IBD overall and persistent IBD (persistent use of medications or hospitalizations throughout the study period) were associated with an increased risk for stroke (RR 1.15, 95% CI 1.04–1.27 and RR 1.55, 95% CI 1.18–2.04, respectively), while IBD in remission (no use of medication or hospitalizations throughout the study period) had a similar relative risk compared to controls [100]. Several meta-analyses and systematic reviews have also shown similar conclusions, especially when related to female patients [93,101].

A population study using the University of Manitoba IBD Epidemiology Database analyzed 8060 patients with IBD compared to a matched cohort of 80,489 controls. The study found that only patients with Crohn’s disease were at an increased risk (1.32, 95% CI 1.05–1.66) for cerebrovascular disease [98]. A population-based nested case-control study within a cohort of 8054 patients with Crohn’s disease matched to 161,078 patients without Crohn’s disease analyzed the odds of ischemic stroke associated with Crohn’s disease. It found that younger patients (less than 50 years old) with Crohn’s disease had an increased risk of ischemic stroke compared to controls (OR 2.93, 95% CI 1.44–5.98) [97]. Therefore, there is evidence for a link between Crohn’s disease specifically and cerebrovascular disease.

### 4.5. Ischemic Heart Disease

Chronic inflammation, which can be found in those with inflammatory bowel disease, can lead to cardiovascular disease, specifically ischemic heart disease and heart failure, by contributing to the pathogenesis of atherosclerosis and the risk of thrombotic events [102]. Several studies have noted an increased risk of ischemic heart disease in those with inflammatory bowel disease [98,100,103]; however, some studies only see this increased risk in some IBD populations with other characteristics, suggesting that there may be secondary risk factors that are contributory [103] (Table 2).

In a cohort study of 20,795 patients with IBD, Kristensen et al. investigated the risk of myocardial infarction, stroke, and cardiovascular death. The patients with IBD were matched by age and sex to 199,978 controls. The study analyzed patients with IBD overall, those with flares or persistent IBD (defined as those with continued corticosteroid prescriptions or IBD hospitalizations during the study period), and those in remission (discontinued use of medications or no hospitalizations during the study period). It found that, overall, those with IBD had an increased risk of myocardial infarction (RR 1.17, 95% CI 1.05–1.31) and cardiovascular death (RR 1.35, 95% CI 1.25–1.45). The relative risks increased for myocardial infarction when considering flares (1.49, 95% CI 1.16–1.93) and persistent IBD (2.05, 95% CI 1.58–2.65). Additionally, in remission periods compared to controls, the relative risk of myocardial infarction and cardiovascular death was similar, suggesting that the severity of the disease played a role in the risk of ischemic heart disease [100].

Another longitudinal cohort study analyzed coronary artery disease in 356 patients with IBD matched to 712 controls. This study also found an increased incidence of coronary artery disease in those with patients with IBD compared to the control group. The unadjusted hazard ratio was 2.85 (95% CI 1.82–4.46) for developing coronary artery disease in the patients with IBD group. It also concluded that the patients with IBD in the study had a lower burden of other risk factors (hypertension, diabetes, dyslipidemia, and obesity, *p* < 0.01 for all risk factors), and thus IBD was a large contributing factor in the development of coronary artery disease in these patients with the adjusted hazard ratio considering these factors being 4.08 (95% CI 2.49–6.70) [104].

A study of 17,487 patients with IBD and 69,948 healthy controls found that patients with IBD overall did not have an increased risk of ischemic heart disease compared to controls but found that women over 40 years old with IBD had a higher risk of myocardial infarction (HR = 1.6, *p* = 0.003) with the risks between Crohn’s disease and ulcerative colitis being similar. This study concluded that not all patients with IBD were at an increased risk of ischemic heart disease, but that certain groups who suffer from IBD may be [103].

Ultimately, there is evidence of an increased risk of ischemic heart disease for those with IBD [98,100,103]. However, not all studies have made this conclusion, so more studies and meta-analyses should be done to continue to assess this relationship. IBD in some specific groups or the presence of other risk factors may lead to greater risk than IBD alone [103].

### 4.6. Mesenteric Ischemia

Of all the thromboembolic events studied with IBD, ATE is less studied than VTE, and mesenteric ischemia is one of the least recognized complications [105]. However, several emerging studies have linked IBD to mesenteric ischemia.

Patients with IBD and/or chronic constipation were compared to control individuals without these conditions in a population-based case-control study nested in a cohort study to analyze the incidence and risk of intestinal ischemia in these populations. It was found that those with IBD have a higher odds ratio of ischemic colitis (4.2 95% CI 0.5–38.4), but no association was found with acute mesenteric ischemia [106]. A cross-sectional study analyzed the Nationwide Inpatient Sample database to compare hospitalized patients discharged with a diagnosis of IBD to hospitalized patients discharged without this diagnosis (control). It reported an adjusted odds ratio of 3.4 (95% CI 2.9–4.0) for an association of IBD with mesenteric ischemia. With a smaller CI in this study, a stronger connection was found between IBD and mesenteric ischemia in this study [81].

An additional study found mesenteric infarction to be a cause of abdominal pain in patients with ulcerative colitis, thus stressing the importance of including mesenteric ischemia in a differential diagnosis for patients with IBD presenting with abdominal pain. This study outlined four cases of patients with ulcerative colitis and abdominal pain who also were diagnosed with mesenteric colitis. One patient presented with abdominal pain before being diagnosed with ulcerative colitis, two presented with the pain at the time of diagnosis, and one presented after diagnosis [68]. Two additional case reports outline patients presenting with abdominal pain. In both cases, the patients were diagnosed with Crohn’s disease and were also found to have mesenteric atherosclerosis with mesenteric artery thrombosis in one patient [107] and a thrombus in the superior mesenteric artery in the second patient [105]. Thus, this complication should be considered when patients with IBD present with abdominal pain.

## 5. Treatment for IBD and Its Effect on Thromboembolic Risk

The main objective of the treatment of IBD is to reduce symptoms and maintain remission [108,109]. Various factors must be considered to tailor the treatment for individual patients. Before initiating treatment, appropriate medication is determined by activity, distribution, severity (mild, moderate, severe, and fulminant), and response to previous treatment [110,111,112,113]. For induction of remission for mild to moderate active ulcerative colitis (UC), 5-ASA (5-aminosalicylic acid) or mesalamine has been the drug of choice [108,114,115]. Corticosteroids have been used for moderate to severe UC or patients who have failed the therapy with 5-ASA [109,110,114,116]. In the case of steroid-dependent ulcerative colitis, azathioprine and mercaptopurine be effective [114,117,118]. Biologics (e.g., Infliximab) is considered in patients who failed corticosteroids and/or immunomodulators [116,119,120]. With an increased risk of thromboembolism in patients with IBD, the risk of VTE needs to be considered with the treatment of IBD.

In patients with IBD, platelets are activated and form aggregate [114]. An increasing number of abnormal platelet function contributing to the inflammation and pathophysiology of IBD have been reported [115,116,117]. Abnormal platelet function is observed through an increased level of chemokine RANTES in patient IBD [118,119]. With the activation of the platelets, it has been found to increase the risk of thromboembolism [90]. To induce and maintain remission in mild to moderate UC, 5-ASA is used as the first line of treatment [120]. 5-ASA has been shown to lower the levels of RANTES in the plasma of patients with IBD compared to control group without IBD [119]. 5-ASA inhibits platelet activation and might reduce thromboembolism; however, there is no specific study assessing the risk of VTE among the patients using 5-ASA. In a small study with a group of 26 patients, 5-ASA inhibited the platelet activation by thrombin (*p* ≤ 0.02) [121]. Additionally, a randomized controlled trial assessing the safety and efficacy of 5-ASA on 206 patients with the active UC did not report any VTE complications [122]. Although more studies of 5-ASA on the risk are needed, it suggests that 5-ASA may reduce the risk of VTE in patients with IBD.

Hypercoagulation has been observed in patients using corticosteroids or adrenocorticotrophic hormone (ACTH) [123]. A case-control study with 38,765 patients with VTE reported that the use of systemic glucocorticoids has the greatest risk of VTE (Incidence rate ratio (IRR) 2.31; 95% CI, 2.18–2.45) and corresponds to 11 VTE cases per 1000 new users of the medication each year [124]. A study by Higgins et al. analyzed that 335 VTE cases were found within 12 months among the 15,100 patients with IBD. It reported that the absolute rate of VTE was 2.25% (296 of 13,165) for corticosteroid therapy. Patients taking the corticosteroids were at risk of developing VTE five times more than those who were treated with only biologics [58]. These studies suggest that there is a higher risk of VTE with the use of corticosteroids.

There has not been a reported incidence of thromboembolism with immunomodulatory therapy. In a study of 3391 Spanish patients with IBD, there is no report of VTE with the use of azathioprine and mercaptopurine after a median follow-up of 44 months [125]. In a prospective study looking at the tolerability of thiopurines, after the median follow-up of 32 months, no VTE events were reported from the analysis of 253 patients [126]. Similarly, a VTE event was not reported from the examination of 174 patients with Crohn’s disease in the study of tolerability of methotrexate after thiopurine therapy [127]. These studies indicate that there may be no potential risk of VTE with immunomodulators.

Tumor necrosis factor (TNF) is a pro-inflammatory cytokine that has been known to activate coagulation [128,129]. Therefore, infliximab may reduce the risk of VTE. Anti-TNF therapy, administered in 452 hospitalizations out of 1048 hospitalizations, was associated with significantly lowering the risk of thromboembolism (OR = 0.201; 95% CI 0.041–0.994; *p* = 0.049) [130]. In a prospective study, 78 out of 103 patients with IBD that have responded to infliximab therapy have shown no increased risk for thrombosis. During the study, patients on infliximab did not develop VTE, while one patient who stopped infliximab and received corticosteroid treatment developed VTE in 1-year follow-up [131]. In another prospective study, it is suggested that infliximab may reduce the risk of VTE in patients with IBD [132]. Clot lysis profiles of patients on infliximab have been reported to decrease or normalize [131,132]. As a result, infliximab may be an effective treatment in reducing the risk of thromboembolism.

## 6. Treatment Recommendations for IBD to Reduce Risk

About 50% of VTE events arise from hospitalization, trauma, and surgery [133]. The risk of VTE increases with factors such as hospital admission, surgery, cancer, and IBD [134,135]. To lower the risk, lower molecular weighted heparin is generally recommended as prophylaxis to hospitalized patients with and without IBD [136,137].

At the time of active IBD, patients are at risk of developing VTE [90]. In a cohort study, the risk of VTE is reported to be higher compared to the time when the disease is inactive [78]. Additionally, there is an increased risk of recurrence in patients with IBD [84]. Although the risk of thrombosis increases with outpatient flares, the absolute risk is low [78]. VTE prophylaxis is not recommended during IBD flare in patients with no history of VTE [138]. However, thromboprophylaxis is recommended for moderate to severe IBD flares with a history of VTE [139].

Among patients with IBD, hospitalization increases the risk of VTE. It has been reported that VTE-associated mortality in patients with IBD is greater compared to patients without IBD [80]. With this risk in patients with IBD, pharmacological thromboprophylaxis is recommended when patients are admitted to the hospital [140]. In a study, the use of low-molecular-weight heparin dalteparin had reduced the thromboembolic events by 45% (Relative risk, 0.55; 95% CI, 0.38 to 0.80; *p* = 0.0015) compared to a placebo group [141]. A meta-analysis showed that heparin and low-molecular-weight heparin used for patients hospitalized with IBD flare up has no difference in adverse effects when compared to controls [142]. Thromboprophylaxis treatment during hospitalization is also associated with a reduced risk of VTE after discharge [92]. Additionally, heparin has been shown to have anti-inflammatory effects and tissue repair properties [143]. More evidence is needed, but studies have proposed some benefits of thromboprophylaxis.

While temporal trends of COVID-19 cases in patients with IBD across 73 countries parallel the epidemiological pattern of COVID-19 in the general population [144], it is important to note the immunosuppressive nature of the drugs used in IBD treatment in patients with concurrent IBD and COVID-19 infection [145]. Although many patients with IBD are prescribed immunosuppressants, which can negatively affect their response to a COVID-19 infection, it is recommended that they continue the therapy if they have no symptoms and are not infected by COVID-19. For patients with IBD who test positive for COVID-19 and have symptoms, the recommendation is to withhold IBD therapy until they recover [146]. Additional prophylactic anticoagulation therapy is recommended to discharged patients with positive COVID-19 as COVID-19 is associated with a hypercoagulable state and thus predisposes the patient to venous thromboembolism [147].

## 7. Conclusions

Thromboembolic events are associated with a substantial increase in mortality and morbidity. The inflammatory response in patients with IBD leads to a hypercoagulable state significantly increasing the risk of thromboembolic events. Several factors associated with IBD increase the risk of thromboembolic events such as the severity of disease, hospitalization, surgery, and corticosteroids. A thorough history must be conducted to assess the individual patients’ risk of thromboembolism.

Guidelines indicate the use of thromboembolic events prophylaxis for all patients hospitalized with IBD flare up if no contraindication is present. For the prophylaxis of thromboembolic events, either heparin or low molecular weight heparin should be considered. However, it is unclear whether thromboembolic events prophylaxis should continue after discharge or not especially in patients with active disease. Future studies need to consider a risk-based model to assess the benefit and risk of thromboembolic events prophylaxis amongst patients with the highest risk. The risk assessment should be based on patients’ demographic information, medical history, medications, and family history. Lastly, further guidelines are required for the management of ambulatory patients with active IBD disease as these patients are at a high risk of developing thromboembolic events. Future studies need to focus on the type of anticoagulation and duration in patients who develop thromboembolic complications secondary to IBD.

Additionally, the pathophysiology of thromboembolic events in patients with IBD needs to be further investigated. A better understanding of the pathophysiology may allow for the recognition or development of biomarkers to sensitively assess the risk of VTE and guide management.

Although guidelines recommend the use of thromboembolic prophylaxis in patients admitted for IBD flare up, it is not widely used due to concerns about bleeding with anticoagulation and the lack of awareness of the increased risk of thromboembolic complications. We hope to increase clinician recognition of thromboembolic events in patients with IBD.

## Figures and Tables

**Table 1:** Summary of studies reporting the risk of VTE in patients with IBD.

Author (Year)	Findings
Yuhara (2013) [85]	This study found an RR of 2.2 (95% CI 1.83–2.65) when comparing the risk of VTE among subjects with and without IBD with similar results after adjusting for obesity and smoking.
Fumery (2014) [73]	The overall risk of VTE in an IBD population was increased by 96% in this study compared to the general population, (RR = 1.96, 95% CI = 1.67–2.30) with no statistical difference between UC and CD subgroups.
Papay (2013) [86]	This study found that 90% of VTE’s were DVT’s and PE’s among patients with IBD.
Bernstein (2007) [75]	VTE occurrence among hospitalized patients with IBD was significantly higher compared to hospitalized patients without IBD (IRR: 4.5 (UC) and 9.6 (CD)).
Novacek (2010) [84]	The probability of recurrence of VTE 5 years after cessation of anticoagulant medication was elevated among patients with IBD in comparison to patients without IBD (33.4%; 95% confidence interval [CI]: 21.8–45.0 vs. 21.7%; 95% CI: 18.8–24.6; *p* = 0.01).
Grainge (2010) [78]	This study found an RR of 3.0 (CI 1.7–6.3) when comparing VTE occurrence in hospitalized patients with IBD to those without.
Nguyen (2008) [80]	This study reported a significantly higher risk of VTE in patients with IBD discharges compared to non-IBD discharges. (OR 1.85 for UC and OR 1.48 for CD). Additionally, VTE was associated with increased mortality, longer hospital stays (by an average of 5 days) and a higher healthcare cost ($47,515 vs. $21,499) (OR 2.5)

**Table 2:** Summary of studies reporting the risk of ischemic heart disease in patients with IBD.

Author	Findings
Kristensen (2013) [100]	Patients with IBD with flares or persistent disease had an increased risk (RR 1.17, 95% CI 1.05–1.31) of myocardial infarction compared to control patients. Patients with IBD with flares or persistent disease had an elevated risk (RR 1.35, 95% CI 1.25–1.45) of cardiovascular death compared to control patients. In patients with ongoing IBD flares this study reported an increased risk of myocardial infarction (RR 1.49, 95% CI 1.16–1.93) when compared to control patients. In patients with persistent IBD this study reported an elevated risk of myocardial infarction (RR 2.05, 95% CI 1.58–2.65) when compared to control patients.
Ha (2009) [103]	This study found that patients with IBD overall did not have an elevated risk of ischemic heart disease compared to controls but found that women over 40 years of age with IBD had a higher risk of myocardial infarction (HR = 1.6, *p* = 0.003) with similar risk between Crohn’s disease and ulcerative.
Yarur (2011) [104]	The incidence of coronary artery disease was significantly elevated in patients with IBD (HR 4.08, CI 2.49–6.70) compared to control patients even after adjusting for concurrent risk factors.
